# 

**Published:** 1913-05-25

**Authors:** 


### THE HOSPITAL

The Modern Newspaper of Administrative Medicine and Institutional Life.

No. 1,404. Vol. LIV. Sunday, May 25, 1913. Price One Penny.

[Registered as a Newspaper]

